# Feasibility of Recovery Assessment Scale – Domains and Stages (RAS-DS) for everyday mental health practice

**DOI:** 10.3389/fpsyt.2024.1256092

**Published:** 2024-02-09

**Authors:** Shivani Ramesh, Justin Newton Scanlan, Anne Honey, Nicola Hancock

**Affiliations:** Centre for Disability Research and Policy, Faculty of Medicine and Health, University of Sydney, Sydney, New South Wales, Australia

**Keywords:** RAS-DS, outcome measure, feasibility, recovery, self-rated outcomes

## Abstract

**Introduction:**

Routine use of self-rated measures of mental health recovery can support recovery-oriented practice. However, to be widely adopted, outcome measures must be feasible. This study examined the feasibility of Recovery Assessment Scale – Domains and Stages (RAS-DS) from the perspectives of mental health workers.

**Method:**

Mental health workers who had previously sought permission to use RAS-DS (n=58) completed an online survey that explored three aspects of feasibility: practicality, acceptability and applicability.

**Results:**

The highest-rated feasibility items related to applicability, or usefulness in practice, with over 90% of participants reporting that RAS-DS helps “promote discussion” and covers areas that are “meaningful to consumers”. Acceptability items indicated that the purpose of RAS-DS is clear but length was an issue for some participants. At a practical level, RAS-DS was seen as easy to access but training was seen by many as necessary to ensure optimal use.

**Conclusion:**

Results suggest potential usefulness of RAS-DS as a routine outcome measure and identify aspects that can be addressed to further enhance feasibility including provision of training materials and opportunities, wide-reaching promotion of its use as a collaborative tool, and further investigation of issues around instrument length.

## 1 Introduction

Over the last three decades, the notion of recovery and adoption of recovery-oriented practice has gained prominence in mental health services (1, 2). Shifting from a dominant focus on symptom amelioration (3), recovery refers to a “deeply personal, unique process of changing one’s attitudes, values, feelings, goals, skills and/or roles - a way of living a satisfying, hopeful and contributing life, even with limitations caused by illness” (4, p. 527). Recovery-oriented practice is grounded in the belief that individuals experiencing mental illness can recover. Practice should embody a collaborative and person-centred approach that fosters self-determination, choice, and hope (5).

Concurrent with shifts towards recovery-oriented practice, mental health services are increasingly expected to evaluate service delivery through use of routine outcome measures (6, 7). Routine outcome measurement was also introduced to support mental health workers in clinical decision-making and engaging consumers in care-planning (6, 8). Current routine outcome measures mandated in countries such as Australia include a mixture of clinician-rated (e.g., Health of the Nation Outcome Scales (9)) and self-rated (e.g., Behaviour and Symptom Identification Scale-32 (10)) measures that focus on symptomology, hospitalisation rates and level of functioning (7). However, in the context of moving towards recovery-oriented practice, Lakeman (11) and Happell (12) argued that mandating routine use of such symptom-focused measures does little to inform recovery-oriented approaches and is not aligned with what is meaningful to consumers in their recovery. Indeed, this may create barriers to recovery-oriented practice (13).

The ability to assess and measure recovery-focused outcomes from the consumer’s perspective supports the adoption of recovery-oriented practice (14, 15). Using self-rated recovery measures presents opportunities for workers to focus on what is important to individual consumers, and actively engage consumers through collaborative goal-setting and care-planning, all fundamental to recovery-oriented practice (16, 17). While mandating completion of a recovery measure by consumers would be antithetical to the choice and autonomy inherent in the concept of recovery, routinely offering it and explaining the benefits is valuable in developing recovery-oriented services.

Consequently, efforts have been made to establish psychometrically sound recovery measures that can be used for routine outcome measurement (18–20). However, none of these measures have been comprehensively examined for their feasibility, with typically only brevity explored. To support the uptake of self-rated recovery measures in routine practice, it is important that measures are not only psychometrically sound but also feasible. For example, low or variable completion rates of routine outcome measures are common (6, 7, 21) and these are often related to feasibility issues such as completion times, scoring complexity, unmet training needs, and, in particular, limited valuing of the measures by workers (22–26).

RAS-DS is a 38-item self-rated recovery measure that encompasses four recovery domains: “Doing things I value” (functional aspects), “Looking forward” (psychological aspects), “Mastering my illness” (symptom management), and “Connecting and belonging” (social aspects). Users rate items on a four point scale (1 untrue, 2 a bit true, 3 mostly true, 4 completely true). It was developed from the Recovery Assessment Scale (RAS) (27), with revised structure and items to address identified problems with RAS measurement properties (28). Formal training is not required to administer RAS-DS and a user-manual can be readily accessed online to guide workers (29). Items are all positively phrased so that a total recovery score can be obtained by simply adding all items and domain scores are obtained by averaging item scores in each domain. RAS-DS has demonstrated good reliability, validity, and sensitivity to change (30, 31). Beyond measuring outcomes, however, RAS-DS was designed as a tool to guide workers’ collaborative practice with consumers (29). It has been translated into 18 languages and uptake has been widespread across 26 countries. While these features make it a promising candidate for a routine outcome measure, little work has been done to establish its feasibility. Previous studies have suggested that it was relatively quick and easy to use and contributed towards goal-setting and the therapeutic alliance (31, 32). However these studies touched on feasibility issues in the context of broader analysis of measurement properties. Given the barriers to implementing routine outcome measures, a more comprehensive assessment of the feasibility of RAS-DS is required. The aim of this study was to explore the feasibility of RAS-DS in detail from the perspectives of mental health workers.

## 2 Methods

### 2.1 Study design

This descriptive study used surveys with RAS-DS users to collect both quantitative and qualitative data to gain a nuanced understanding of participants’ perspectives of the feasibility of RAS-DS (33). Ethical approval was obtained from The University of Sydney Human Research Ethics Committee (approval number 2019/921).

### 2.2 Measuring feasibility

One of the most influential feasibility frameworks used in mental health services was developed by Andrews et al. (34). This framework proposed that a “feasible” measure is *practical*, *acceptable*, and *applicable* to consumers and workers. A *practical* measure imposes minimal cost. Further, administering, scoring, and interpreting results should be simple, with instructions provided and little training needed. An *acceptable* measure is brief and user-friendly, for example, the format and language should be easy for consumers to understand. Lastly, an *applicable* measure addresses outcomes aligned with consumer priorities and facilitates appropriate treatment decisions. In recovery-based practice, facilitating appropriate treatment decisions requires consumer involvement and understanding between worker and consumer. To be applicable, a measure should also be multi-dimensional to enable specific domains to be illuminated, and able to be meaningfully aggregated for management requirements. This framework has been utilised in previous studies (35, 36) to evaluate the feasibility of potential routine outcome measures. In addition to aspects covered within the Andrews et al. (34) framework, some additional feasibility considerations have also been suggested. These include cultural appropriateness, capacity to promote discussions between consumers and workers (36), flexibility in administration options, and ability to provide information otherwise unavailable, contributing towards workers’ understanding of consumers (37). Therefore, for this study, the Andrews et al. (34) framework was expanded upon to include these additional aspects. The updated feasibility framework is summarised in **Table 1**.

**Table 1:** Updated feasibility framework adopted in this study.

Feasibility Domain	Considerations
Practicality	Minimal training requirements ^a^ Easy to score (i.e. simple and quick calculation of totals) ^a^ Easy to interpret results (i.e. the meaning of item and total scores is clear) ^a^ Easy to access ^a^ Low cost ^a^
Acceptability	Brief (i.e., not too many questions and items) ^a^ Purpose is clear and relevant to consumers ^a^ Wording is easy to understand for consumers ^a^ Culturally appropriate ^b^ Flexible administration (i.e., consumers are able to choose whether to complete the tool on their own or with workers) ^c^
Applicability	Covers areas or concepts that are meaningful and important to consumers ^a^ Promotes discussion ^b^ Gives workers clearer understanding of consumer perspectives ^c^ Helps consumers participate in decision-making and treatment planning and tracking outcomes ^a^ Encourages recovery-oriented practice ^a^ Multi-dimensional ^a^ Able to be aggregated (i.e., meaningful totals can be calculated) ^a^

^a^Adapted from Andrews, Peters (34). ^b^Adapted from Siggins Miller Consultants (36). ^c^Adapted from Slade, Thornicroft (37).

### 2.3 Participants

Eligible participants were workers currently or previously using RAS-DS. Given the absence of a comprehensive sampling frame, convenience sampling was used. RAS-DS information specifies that, while the tool is freely available, the author’s permission is required for organisations to use it. The author keeps a database of people who have requested this permission and have agreed to receive information pertaining to it. Invitations were sent to the 183 individuals on this database. In recognition that that many people and organisations do not seek permission and that other individuals at each organisation will use RAS-DS, the invitation encouraged potential participants to distribute the invitation to other colleagues who had used RAS-DS. The email included a Participant Information Statement and the link to the survey.

### 2.4 Data collection

The survey instrument was developed for this study and pilot-tested with two RAS-DS users from Australia and Canada. It collected information on: a) participants’ demographics and practice context, b) current or previous use of RAS-DS; and c) feasibility aspects of RAS-DS using items developed from the updated feasibility framework (**Table 1**). No items were included around RAS-DS being low cost, multidimensional and able to be aggregated given that these are objective features of RAS-DS (which is cost-free, is arranged around four domains and has demonstrated measurement properties that support aggregation) (31).

Throughout the survey, participants were given the option to explain or comment on their responses, and they provided free-text responses to questions about most and least useful aspects of RAS-DS. Current RAS-DS users were also asked further questions regarding their RAS-DS use (e.g., why and how RAS-DS was used, frequency of use and whether RAS-DS helped them be more recovery-oriented in their practice).

Study data were collected and managed using the REDCap electronic data capture tool hosted at The University of Sydney (38).

### 2.5 Data analysis

Frequencies, medians and interquartile range values were calculated for fixed choice items. Qualitative data from open-ended questions ranged from short phrases to paragraphs. Constant comparative analysis was used to thematically analyse this data (39). Due to conceptual overlap in responses, data from different questions were analysed together to compile and quantify the overall frequency of themes. Initial coding began with reading each response and identifying and labelling underlying concepts (39). With each response, data were compared with existing codes to check if they conveyed similar meanings. New concepts were labelled as new codes. Progressively, codes were compared to one another and grouped into higher-level categories if conceptual similarity existed (39). To enhance trustworthiness, the authors SR and NH independently coded the full data set and subsequently discussed their interpretations and categorisations before reaching consensus. Counts were made of participants reporting each thematic category.

## 3 Results

### 3.1 Participants

The survey was commenced by 79 participants, however 20 responses were excluded as they had insufficient usable data or the participant (n=1) did not meet the inclusion criteria as they did not provide consent for participation. The final sample size was 58. All participants provided informed consent. Demographic characteristics of participants are summarised in **Table 2** and their described use of RAS-DS are presented in **Table 3**.

**Table 2:** Demographic characteristics (N = 58).

Characteristic	n	%
Gender		
Female	47	81.0
Male	10	17.2
Prefer not to say	1	1.7
Age		
20 to 29 years	8	13.8
30 to 39 years	14	24.1
40 to 49 years	20	34.5
50 to 59 years	11	19.0
60 to 69 years	5	8.6
Length of time working in mental health		
Less than 1 year	1	1.7
1 to 5 years	15	25.9
6 to 10 years	15	25.9
11 to 20 years	9	15.5
Over 20 years	17	29.3
Prefer not to say	1	1.7
Professional qualification ^a^		
Occupational therapy	14	24.1
Nursing	12	20.7
Counselling	11	18.9
Consumer worker/peer worker	10	17.2
Psychiatry	8	13.8
Psychology	8	13.8
Social work	1	1.7
Other (e.g., research, social policy consultancy)	6	10.3
Practice settings ^a^		
Mental health services – Community based	38	65.5
Mental health services – Acute inpatient/hospital	17	29.3
Mental health services – Inpatient rehabilitation	11	19.0
Drug and Alcohol/Addiction services	3	5.2
Others (e.g., forensic, veteran or suicide prevention services and university academic services)	8	14.8
Not applicable	1	1.6
Age groups of consumers worked with ^a^		
Children (0 to 12)	1	1.7
Adolescents and Youth/Early intervention services (approx. 12 to 25)	16	27.6
Adults (18 to 65)	51	87.9
Older adults (65 and over)	14	24.1
Country of practice		
Australia	28	48.3
Other (United States (n=6); United Kingdom (n=5); Indonesia (n=3); Canada (n=2); Iceland (n=2); India (n=2); Singapore (n=2); Chile, Egypt, Netherlands, New Zealand, Philippines, Switzerland, Thailand, Turkey (n=1 each))	30	51.7

^a^Participants could select more than one qualification, practice setting or consumer age-group; therefore, totals may add to more than 100%.

**Table 3:** Use of RAS-DS (n = 49).

Characteristic	n	%
RAS-DS use		
Current user Previous user^a^	49 9	84.5% 15.5%
Frequency of use of RAS-DS with each consumer		
Monthly Every 3 to 6 months Yearly Once only	10 30 4 5	20.4 61.2 8.2 10.2
Proportion of consumers offered RAS-DS		
Over 90% Between 75% and 90% Between 50% and 90% Less than 50%	24 6 4 15	49.0 12.2 8.2 30.6

^a^The remainder of the table only includes the data from participants who were currently using RAS-DS (n = 49).

### 3.2 Feasibility of RAS-DS

#### 3.2.1 Practicality

Summary results for practicality questions are presented in **Table 4**. Responses to the practicality questions indicate that, while most people found it practical, a significant minority indicated that training and support was, or would be beneficial, especially around interpretation. A large majority of participants saw RAS-DS as easy to access and score (86.6% and 81.5% respectively). While still a majority, fewer agreed that the results were easy to interpret (66.7%) and that minimal training was required (62.3%).

**Table 4:** Practicality ratings and scores.

Practicality Item	n^a^	Agree^b^ % (n)	Disagree^c^ % (n)	Median (IQR^d^)
Easy to access	52	86.6 (45)	1.9 (1)	4.00 (4.00 – 5.00)
Easy to score	54	81.5 (44)	9.3 (5)	4.00 (4.00 – 5.00)
Easy to interpret results	54	66.7 (36)	9.3 (5)	4.00 (3.00 – 4.00)
Minimal training required	53	62.3 (33)	13.2 (7)	4.00 (3.00 – 4.00)

^a^‘I don’t know’ responses treated as missing data. ^b^Combined “Agree” and “Strongly agree” responses. ^c^Combined “Disagree” and “Strongly disagree” responses. ^d^IQR, Interquartile range.

Participants’ qualitative comments, both in relation to their ratings for these items and in response to other open-ended questions, provide additional detail about their perceptions of its practicality. In terms of ease of access, positive comments often referenced RAS-DS being freely available online, however some participants seemed unaware of this and mentioned having accessed RAS-DS through the author or their workplace. The one participant who disagreed that RAS-DS was easy to access described “having to print it out on paper is a pain” (P44) and three others noted that “an app would be immensely useful” (P34). A number of people who found the RAS-DS very easy to score indicated that they had used automatic scoring provided by the authors in Excel or provided by their employers, while some who gave a low rating for ease of scoring described manual scoring as time consuming. Of the 18 people who commented about their rating, the most common comments (n=7), made by people with quite different ratings, centred around the meaning of scores being highly individualised and the need to interpret RAS-DS in conversation or “consultation with the consumer” (P13) themselves.

Seventeen people (29%) commented on the need for training. Four (7%) characterised RAS-DS as “self-explanatory”. However, thirteen (22%), with varying ratings of agreement on this question, believed that some training was required, especially for workers to meaningfully integrate RAS-DS and its results into practice and for people with less clinical training and experience (e.g., “people need to know how to use it properly as a tool to engage conversations with people, instead of just get them to tick boxes” [P44]).

Five participants (9%) noted that practical issues were sometimes related, not to RAS-DS per-se, but to their own services, for example the time they had available, lack of platform for documentation of results and lack of awareness of the tool amongst different teams. (e.g., “It depends on the time provided in the clinic. When I have a lot of patients, there were limited time to use the RAS-DS” (P75)).

#### 3.2.2 Acceptability

Summary results for acceptability questions are presented in **Table 5**. A large majority of people agreed that the RAS-DS had a clear and relevant purpose, that items were easy to understand and that consumers could choose method of completion. The lowest rated items in any domain were those relating to the length of RAS-DS.

**Table 5:** Acceptability ratings and scores.

Acceptability Item	n^a^	Agree^b^ % (n)	Disagree^c^ % (n)	Median (IQR^d^)
Not too long for clients^e^	54	51.8 (28)	25.9 (14)	4.00 (2.00 – 4.00)
Does not have too many items^e^	54	48.1 (26)	22.2 (12)	3.00 (3.00 – 4.00)
Purpose is clear and relevant	53	90.6 (48)	5.7 (3)	4.00 (4.00 – 5.00)
Wording of items is easy to understand	53	81.1 (43)	7.5 (4)	4.00 (4.00 – 4.00)
Culturally appropriate	53	69.8 (37)	1.9 (1)	4.00 (3.00 – 4.00)
Consumers are able to choose how to complete RAS-DS	54	85.2 (46)	7.4 (4)	4.00 (4.00 – 4.00)

^a^54 participants completed this section. Differences in ‘n’ due to ‘I don’t know’ responses being treated as missing data. ^b^Combined “Agree” and “Strongly agree” responses. ^c^Combined “Disagree” and “Strongly disagree” responses. ^d^IQR, Interquartile range. ^e^For simplicity of comparison, negatively-worded items are re-phrased and reverse scored for the purpose of table.

A total of 32 participants (55%) commented about the length of RAS-DS. While six (11%) characterised is as “short” (P27) or “brief” (P19), 12 participants (22%) indicated that RAS-DS was “a bit long” (P32), or had too many items. For example, one explained that “our patients complete the tool but will do better with a shorter version” (P71). In contrast, 5 participants (9%) explained that the length was less important than the potential benefits of RAS-DS or that the items were necessary to explore recovery holistically. P1, for example thought that RAS-DS “needs to have the level of detail as it gets clients thinking of many aspects of recovery”, while P34 stated that “there are a few questions, but the fact that they help encourage conversation is important”. Five participants (9%) explained that the appropriateness of the length depended on the consumer, for example the acuteness of their symptoms, their cognition or their literacy. However, four others (7%) described using strategies to address these issues, such as using RAS-DS “one section at a time” (P41) and “assist(ing) (consumers) in completing the form” (P54).

Twenty-six people commented about other acceptability issues in their discussion of most and least helpful features of the RAS-DS or explanation of their usefulness rating. Seventeen of these were positive about the use of language, with 9 (16%) commenting that it was easy for consumers to understand the purpose and “the language is simple to understand” (P2). Nine (16%) appreciated the use of positive language, “a more positively worded set of questions” (P28) and that “it doesn’t feel like a pass/fail thing” (P58). However, seven people (13%) felt that the wording could be abstract or confusing for some consumers, especially those experiencing acute symptoms or cognitive limitations. When discussing least helpful aspects, a small number of participants also brought up issues with specific items (n=4; 7%) and lack of cultural appropriateness (n=2; 4%) (e.g., “It does not include element of recovery specifically related to family, which is important in an Asian context.” (P10)).

#### 3.2.3 Applicability

Summary results for applicability questions are presented in **Table 6**. Applicability was the highest ranked domain of feasibility overall. Of the 45 participants currently using RAS-DS who reported the overall usefulness of RAS-DS, more than 90% reported finding it moderately useful (n=10), quite useful (n=15) or very useful (n=17) overall. Two of those who reported RAS-DS only slightly useful or not useful explained that this was because they had limited understanding/education on how to use RAS-DS. Another said usefulness was limited as few workers in their service were recovery-oriented.

**Table 6:** Applicability ratings and scores.

Applicability Item	n^a^	Agree^b^ % (n)	Disagree^c^ % (n)	Median (IQR^d^)
Areas covered are meaningful and important to the people I work with	54	92.6 (50)	0.0 (0)	4.00 (4.00 – 4.25)
Gives clearer understanding of consumers and their perspectives	53	86.8 (46)	3.8 (2)	4.00 (4.00 – 5.00)
Helps consumers participate in decision making, treatment planning and tracking progress	51	80.4 (41)	5.9 (3)	4.00 (4.00 – 5.00)
Promotes discussion	53	94.3 (50)	5.7 (3)	4.00 (4.00 – 5.00)

^a^54 participants completed this section. Differences in ‘n’ due to ‘I don’t know’ responses being treated as missing data. ^b^Combined “Agree” and “Strongly agree” responses. ^c^Combined “Disagree” and “Strongly disagree” responses. ^d^IQR, Interquartile range. ^e^For simplicity of comparison, negatively-worded items are re-phrased and reverse scored for the purpose of table.

Of the more specific applicability items, the highest-rated items were “The RAS-DS helps promote discussion” and “The areas covered by the RAS-DS are meaningful and important to the people I work with”.

Most participants who responded to the question about whether RAS-DS helped them to take a recovery-oriented approach in practice (40 out of 45, 89%) agreed that it did either to a large (n=17), moderate (n=19) or small (n=4) degree. Three participants were unsure and the two participants who reported “No” explained that they had “always been recovery-oriented” (P56).

Free text responses provided further information about the applicability of RAS-DS. Of the 49 current users, 47 responded to the question asking how they used the results. Five (10%) reported not using them significantly beyond as mandated documentation. For example, P6 stated “I score the RAS-DS, put it in my case notes and don’t look at it again”. Others, however, reported a variety of uses, which are detailed and exemplified in **Table 7**. Forty-six participants commented on applicability aspects of RAS-DS when asked about most useful features, with some providing additional related comments in response to other questions. Because of overlap of these themes with reported use, frequency and examples are also included in **Table 7**.

**Table 7:** Frequency and examples of how RAS-DS results are used and how RAS-DS is regarded as useful.

Themes	Frequency of reported use (% current users)	Reported as useful feature (% all users)	Example quote/s
**Recovery-focused, measuring meaningful aspects of recovery**		22 (38%)	“Broad perspective of personal recovery” (P61) “Focus on quality of life/recovery, not on clinical symptoms” (P19) “Clients are amazed that there is an outcome measure that actually assumes they will/can recover in some way that’s meaningful for them.” (P41)
**Useful/used to develop recovery goals and intervention plans**	21 (43%)	21 (36%)	“Very useful in identifying consumer-defined recovery priorities” (P7)
**Useful/used to monitor recovery progress (individually or collectively)**	21 (43%)	13 (22%)	“Good for pre and post comparison particularly for clients working on non-clinical goals.” (P5) “For program monitoring.” (P10)
**Helpful to support consumers to think about, reflect on and take charge of recovery**		15 (26%)	“Often the idea of recovery is very foreign to them (clients), so this really helps client to start exploring this and thinking this way” (P59) “Emphasis on personal responsibility/ownership for own journey” (P23)
**Useful/used to discuss and understand consumer perspectives on their recovery**	16 (33%)	15 (26%)	“It can help to start conversations regardless of whether a 1 or a 4 is the answer” (P26) “It allows insight to consumers’ experiences and feelings…” (P22)
**Helps worker to stay focused on recovery**		10 (17%)	“Helps keep me recovery focused in my practice” (P1)
**Used to share information with others who support the consumer (e.g., multidisciplinary team, family)**	7 (14%)		“allows for more detailed information to be relayed to the referring GP” (P56) “Clients … find it useful to show family and friends where they’ve made progress” (P2)
**Used to meet research project aims**	3 (6%)		“Research results will be used to understand how recovery relates to support and treatment of people with severe mental illness” (P45)

However, nine participants noted, when asked about least useful features, that the usefulness of RAS-DS was dependent on how the consumer engaged with it, which could be influenced by a variety of features such as consumer’s understanding of their illness, how they were feeling on that day or how much information they wanted to disclose with worker. For example, P6 stated that “It is very dependent on how the person is feeling on the day and as to what they actually want to disclose”.

## 4 Discussion

The purpose of this study was to examine the feasibility of RAS-DS in detail from workers’ perspectives. Overall, results support previous findings that have suggested that RAS-DS is easy to use and valued by consumers and workers for its ability to facilitate goal-setting, prompt discussions about recovery, and track recovery progress (31, 32). This study provides additional information and detail, however, especially about aspects of feasibility that most support routine use and aspects that warrant further consideration.

Applicability was the highest-rated feasibility domain of RAS-DS and a large proportion of participants described applicability aspects as what they found most useful about RAS-DS. This is promising as research has repeatedly demonstrated that workers are more willing to adopt routine measures that contribute to practice (25, 40) and promote discussions that inform care-planning (41–43). Free-text responses also show how RAS-DS can promote recovery-oriented practice. For example, understanding consumer perspectives, stimulating reflections on recovery and actively engaging consumers in goal-setting are seen as critical to ensuring that interventions are person-centred and meaningful to consumers (44). Some participants, however, indicated using RAS-DS in a more service-oriented way, for example, for staff to monitor individual progress or the overall impact of services. While a number of barriers have been found to workers implementing RAS-DS as a recovery planning tool rather than just an outcome measure (45), this study demonstrates its potential for use for many purposes. However, it is clear from the minority of participants who appeared to make little use of the results, that mandating RAS-DS is of little use if workers do not have the time, understanding or the will to use it as designed.

Despite many positive responses, some participants raised concerns regarding the acceptability of RAS-DS. First some participants found it was too long overall, or too long for certain consumers. Although RAS-DS takes most consumers 15 minutes or less to complete (31), this is worth considering given that workers are less likely to use tools they deem too long (22, 40). However, the same studies have concurrently evidenced that workers are more likely to use a tool they believe informs practice (22, 40). In the current study, some participants suggested that the length of RAS-DS was of lesser importance compared with its benefits and that the length was necessary to explore recovery holistically. A shorter measure, although more practical, may be less applicable. Crawford et al. (46) found that compared to shorter tools, consumers valued in-depth tools that examined different aspects of life and facilitated discussions. Therefore, this prompts the question: how brief should a measure be to be practical, whilst still applicable and useful to workers and consumers? Promotion of the benefits of RAS-DS for use as a recovery planning tool rather than just an outcome measure (45) and education around different ways to complete the RAS-DS, such as across multiple sessions, may help to alleviate this perception. However, further exploration of worker and consumer perspectives on how to achieve this balance is warranted.

Related to practicality, many participants believed additional support or training was needed to optimally use RAS-DS and its results to guide practice. The type of training provided on the use of a routine measure can be pivotal in determining whether it is used solely as an administrative tool or as a tool to guide and inform practice (47, 48). For self-rated measures such as RAS-DS, training focused on how they can be used collaboratively with consumers can increase workers’ perceptions of their value and facilitate uptake (22, 49). Although developing a tool that is easy to use with minimal training is critical for uptake (50), further consideration is needed to ensure that RAS-DS is used optimally and as intended. The authors have conducted additional research into the facilitators and barriers to using RAS-DS as more than an outcome measure (45) and, based on these findings, are currently developing an app that will incorporate guidance for workers on the implementation of RAS-DS and interpreting the results as well as support its use for recovery focused conversations and person-centred goal setting. This app will also address other practicality issues by eliminating the need for paper copies and by calculating scores, thus making the RAS-DS easier to use.

Some participants believed the usefulness of RAS-DS was dependent on consumer factors such as their understanding of their illness, mood, or how much they wanted to disclose. Similar criticisms have been reported previously with clinicians expressing concerns that consumers may over or under-represent their experiences and doubting the validity and results of self-rated measures if they did not align with their clinical judgement (51–53). However, considering the deeply personal and unique nature of recovery (4), it is argued that no one apart from consumers themselves can measure their recovery (54). Perhaps, workers should instead view differences in opinions as opportunities to further explore consumer perspectives and to develop shared understandings of consumers’ experiences. Further, consideration should be given to relational issues. Consumers may respond “strategically” if there was mistrust or fear about how their self-ratings would be interpreted and whether it would affect services they received (55, 56). Developing a strong and trusting therapeutic alliance with consumers is key in creating a safe environment for consumers to respond honestly when using self-rated measures (56).

### 4.1 Limitations

This study gathered worker perspectives of RAS-DS. While workers are the “gatekeepers” of the tool and determine whether and how it is used, understanding consumer perspectives about feasibility is essential (13). While previously examined to some degree (31, 32), consumer perspectives should be addressed in more detail in future studies.

A major limitation of this study relates to the use of convenience sampling to recruit participants. It is likely that people who contacted the author for permission to use RAS-DS, were using it at the time of the study (as 85% of respondents were), and chose to respond to a survey about it, would value its use to a greater extent than those who did not. Therefore, results of the study may well be positively skewed and thus not necessarily generalisable to the wider population of mental health workers. Nevertheless, the study suggests aspects of RAS-DS that make it viable as a routine outcome measure and those requiring further consideration.

### 4.2 Conclusion

Notwithstanding the limitations, the findings from this study indicate a potential for RAS-DS to be used as a routine outcome measure of recovery. They also suggest that the RAS-DS has great potential to promote meaningful recovery-focussed discussions with consumers, facilitate collaborative care-planning, monitor consumer recovery progress and support recovery-oriented practice. Although further consideration of length may be warranted, initiatives in progress to address training needs and promote the use of RAS-DS as more than an outcome measure will further enhance its feasibility.

## Data availability statement

The raw data supporting the conclusions of this article will be made available by the authors, without undue reservation.

## Ethics statement

The studies involving humans were approved by The University of Sydney Human Research Ethics Committee. The studies were conducted in accordance with the local legislation and institutional requirements. The participants provided their written informed consent to participate in this study.

## Author contributions

SR: Data curation, Formal Analysis, Investigation, Writing – original draft. JS: Conceptualization, Methodology, Supervision, Writing – original draft. AH: Formal Analysis, Writing – review & editing. NH: Methodology, Supervision, Writing – original draft.

## References

[1] Leamy M Bird VJ Davidson L Williams J. What does recovery mean in practice? A qualitative analysis of international recovery-oriented practice guidance. Psychiatr Serv (2011) 62(12):1470–6. doi: 10.1176/appi.ps.001312011 22193795

[2] Chester P Ehrlich C Warburton L Baker D Kendall E Crompton D. What is the work of recovery oriented practice? A systematic literature review. Int J Ment Health Nurs (2016) 25(4):270–85. doi: 10.1111/inm.12241 27381002

[3] Davidson L O'Connell MJ Tondora J Lawless M Evans AC. Recovery in serious mental illness: A new wine or just a new bottle? Prof Psychology Res Pract (2005) 36(5):480–7. doi: 10.1037/0735-7028.36.5.480

[4] Anthony WA. Recovery from mental illness: the guiding vision of the mental health service system in the 1990s. Psychosocial Rehabil J (1993) 16(4):11–23. doi: 10.1037/h0095655

[5] Farkas M Gagne C Anthony W Chamberlin J. Implementing recovery oriented evidence based programs: identifying the critical dimensions. Community Ment Health J (2005) 41(2):141–58. doi: 10.1007/s10597-005-2649-6 15974495

[6] Roe D Drake RE Slade M. Routine outcome monitoring: an international endeavour. Int Rev Psychiatry (2015) 27(4):257–60. doi: 10.3109/09540261.2015.1070552 26271399

[7] Burgess P Pirkis J Coombs T. Routine outcome measurement in Australia. Int Rev Psychiatry (2015) 27(4):264–75. doi: 10.3109/09540261.2014.977234 25768326

[8] Boswell JF Kraus DR Miller SD Lambert MJ. Implementing routine outcome monitoring in clinical practice: benefits, challenges, and solutions. Psychother Res (2015) 25(1):6–19. doi: 10.1080/10503307.2013.817696 23885809

[9] Wing J Beevor A Curtis R Park S Hadden J Burns A. Health of the nation outcome scales (Honos). Br J Psychiatry (1998) 172(1):11–8. doi: 10.1192/bjp.172.1.11 9534825

[10] Eisen SV Dill DL Grob MC. Reliability and validity of a brief patient-report instrument for psychiatric outcome evaluation. Psychiatr Serv (1994) 45(3):242–7. doi: 10.1176/ps.45.3.242 8188195

[11] Lakeman R. Standardized routine outcome measurement: pot holes in the road to recovery. Int J Ment Health Nurs (2004) 13(4):210–5. doi: 10.1111/j.1445-8330.2004.00336.x 15660588

[12] Happell B. Determining the effectiveness of mental health services from a consumer perspective: part 1: enhancing recovery. Int J Ment Health Nurs (2008) 17(2):116–22. doi: 10.1111/j.1447-0349.2008.00519.x 18307600

[13] Thornicroft G Slade M. New trends in assessing the outcomes of mental health interventions. World Psychiatry (2014) 13(2):118–24. doi: 10.1002/wps.20114 PMC4102275 24890055

[14] Gilburt H Slade M Bird V Oduola S Craig TKJ. Promoting recovery-oriented practice in mental health services: A quasi-experimental mixed-methods study. BMC Psychiatry (2013) 13(1):167–77. doi: 10.1186/1471-244X-13-167 PMC3683325 23764121

[15] Slade M Amering M Farkas M Hamilton B O'Hagan M Panther G. Uses and abuses of recovery: implementing recovery-oriented practices in mental health systems. World Psychiatry (2014) 13(1):12–20. doi: 10.1002/wps.20084 24497237 PMC3918008

[16] Bird V Leamy M Tew J Le Boutillier C Williams J Slade M. Fit for purpose? Validation of a conceptual framework for personal recovery with current mental health consumers. Aust New Z J Psychiatry (2014) 48(7):644–53. doi: 10.1177/0004867413520046 24413806

[17] Law H Morrison A Byrne R Hodson E. Recovery from psychosis: A user informed review of self-report instruments for measuring recovery. J Ment Health (2012) 21(2):192–207. doi: 10.3109/09638237.2012.670885 22559830

[18] Sklar M Groessl EJ O'Connell M Davidson L Aarons GA. Instruments for measuring mental health recovery: A systematic review. Clin Psychol Rev (2013) 33(8):1082–95. doi: 10.1016/j.cpr.2013.08.002 24080285

[19] Penas P Iraurgi I Moreno MC Uriarte JJ. How is evaluated mental health recovery?: A systematic review. Actas Espanolas Psiquiatria (2019) 47(1):23–32. doi: 10.1016/j.eurpsy.2017.01.2145 30724328

[20] Burgess P Pirkis J Coombs T Rosen A. Assessing the value of existing recovery measures for routine use in Australian mental health services. Aust New Z J Psychiatry (2011) 45(4):267–80. doi: 10.3109/00048674.2010.549996 21314238

[21] Pirkis J Callaly T. Mental health outcome measurement in Australia. In: Trauer T, editor. Outcome Measurement in Mental Health: Theory and Practice. Cambridge, UK: Cambridge University Press (2010). p. 15–25.

[22] Callaly T Hyland M Coombs T Trauer T. Routine outcome measurement in public mental health: results of a clinician survey. Aust Health Rev (2006) 30(2):164–73. doi: 10.1071/AH060164 16646765

[23] Gelkopf M Mazor Y Roe D. A systematic review of patient-reported outcome measurement (Prom) and provider assessment in mental health: goals, implementation, setting, measurement characteristics and barriers. Int J Qual Health Care (2021) 34(Supplement_1):ii13–27. doi: 10.1093/intqhc/mzz133 32159763

[24] Hall CL Moldavsky M Taylor J Sayal K Marriott M Batty MJ. Implementation of routine outcome measurement in child and adolescent mental health services in the United Kingdom: A critical perspective. Eur Child Adolesc Psychiatry (2014) 23(4):239–42. doi: 10.1007/s00787-013-0454-2 PMC3973864 23896764

[25] Trauer T Callaly T Herrman H. Attitudes of mental health staff to routine outcome measurement. J Ment Health (Abingdon England) (2009) 18(4):288–97. doi: 10.1080/09638230701879177

[26] Van Wert MJ Malik M Memel B Moore R Buccino D Hackerman F. Provider perceived barriers and facilitators to integrating routine outcome monitoring into practice in an urban community psychiatry clinic: A mixed-methods quality improvement project. J Eval Clin Pract (2021) 27(4):767–75. doi: 10.1111/jep.13457 32790131

[27] Giffort D Schmook A Woody C Vollendorf C Gervain M. Recovery assessment scale. Cambridge, MA: Human Services Research Institute (1995).

[28] Hancock N Bundy A Honey A James G Tamsett S. Improving measurement properties of the recovery assessment scale with Rasch analysis. Am J Occup Ther (2011) 65(6):e77–85. doi: 10.5014/ajot.2011.001818

[29] Hancock N Scanlan JN Bundy AC Honey A. Recovery Assessment Scale - Domains and Stages (Ras-Ds) Manual - Version 3. Sydney, Australia: University of Sydney (2019).

[30] Scanlan JN Hancock N Honey A. The recovery assessment scale – domains and stages (Ras-ds): sensitivity to change over time and convergent validity with level of unmet need. Psychiatry Res (2018) 261:560–4. doi: 10.1016/j.psychres.2018.01.042 29407723

[31] Hancock N Scanlan JN Honey A Bundy AC O’Shea K. Recovery assessment scale - domains and stages (Ras-ds): its feasibility and outcome measurement capacity. Aust New Z J Psychiatry (2015) 49(7):624–33. doi: 10.1177/0004867414564084 PMC4941096 25526940

[32] Hancock N Scanlan JN Kightley M Harris A. Recovery assessment scale - domains and stages: measurement capacity, relevance, acceptability and feasibility of use with young people. Early Intervention Psychiatry (2020) 14(2):179–87. doi: 10.1111/eip.12842 31274238

[33] Bryman A. Integrating quantitative and qualitative research: how is it done? Qual Res (2006) 6(1):97–113. doi: 10.1177/1468794106058877

[34] Andrews G Peters L Teesson M. Measurement of Consumer Outcome in Mental Health: A Report to the National Mental Health Information Strategy Committee. Peters L Teesson M, editors. Sydney: Australia Clinical Research Unit for Anxiety Disorders (1994).

[35] Stedman T Yellowlees P Mellsop G Clarke R Drake S. Measuring Consumer Outcomes in Mental Health: Field Testing of Selected Measures of Consumer Outcomes in Mental Health. Stedman T, editor. Canberra, ACT: Department of Health and Family Services (1997).

[36] Siggins Miller Consultants. Consumer Self-Rated Outcome Measures in Mental Health. Melbourne, Australia: Department of Human Services (2003).

[37] Slade M Thornicroft G Glover G. The feasibility of routine outcome measures in mental health. Soc Psychiatry Psychiatr Epidemiol (1999) 34(5):243–9. doi: 10.1007/s001270050139 10396165

[38] Harris PA Taylor R Thielke R Payne J Gonzalez N Conde JG. Research electronic data capture (Redcap)—a metadata-driven methodology and workflow process for providing translational research informatics support. J Biomed Inf (2009) 42(2):377–81. doi: 10.1016/j.jbi.2008.08.010 PMC2700030 18929686

[39] Charmaz K. Constructing Grounded Theory. 2nd ed. London, United Kingdom: SAGE Publication Inc (2014).

[40] Coombs T Stapley K Pirkis J. The multiple uses of routine mental health outcome measures in Australia and New Zealand: experiences from the field. Australas Psychiatry (2011) 19(3):247–53. doi: 10.3109/10398562.2011.562507 21682624

[41] Willis A Deane FP Coombs T. Improving clinicians' Attitudes toward providing feedback on routine outcome assessments. Int J Ment Health Nurs (2009) 18(3):211–5. doi: 10.1111/j.1447-0349.2009.00596.x 19490232

[42] Guthrie D McIntosh M Callaly T Trauer T Coombs T. Consumer attitudes towards the use of routine outcome measures in a public mental health service: A consumer-driven study. Int J Ment Health Nurs (2008) 17(2):92–7. doi: 10.1111/j.1447-0349.2008.00516.x 18307597

[43] Unsworth G Cowie H Green A. Therapists’ and clients’ Perceptions of routine outcome measurement in the Nhs: A qualitative study. Counselling Psychother Res (2012) 12(1):71–80. doi: 10.1080/14733145.2011.565125

[44] Slade M Bird V Boutillier CL Farkas M Grey B Larsen J. Development of the refocus intervention to increase mental health team support for personal recovery. Br J Psychiatry (2015) 207(6):544–50. doi: 10.1192/bjp.bp.114.155978 26450586

[45] Honey A Hancock N Scanlan JN. Staff perceptions of factors affecting the use of Ras-ds to support collaborative mental health practice. BMC Psychiatry (2023) 23(1):Article 500. doi: 10.1186/s12888-023-04996-2 37438725 PMC10337171

[46] Crawford MJ Robotham D Thana L Patterson S Weaver T Barber R. Selecting outcome measures in mental health: the views of service users. J Ment Health (2011) 20(4):336–46. doi: 10.3109/09638237.2011.577114 21770782

[47] Rye M Rognmo K Aarons GA Skre I. Attitudes towards the use of routine outcome monitoring of psychological therapies among mental health providers: the Ebpas–rom. Administration Policy Ment Health Ment Health Serv Res (2019) 46(6):833–46. doi: 10.1007/s10488-019-00968-5 31485816

[48] Edbrooke-Childs J Wolpert M Deighton J. Using patient reported outcome measures to improve service effectiveness (Upromise): training clinicians to use outcome measures in child mental health. Administration Policy Ment Health Ment Health Serv Res (2016) 43(3):302–8. doi: 10.1007/s10488-014-0600-2 PMC4831995 25331446

[49] Logan J Wheeler A. Outcome measurement in Australian non-government organisations: A descriptive study of recovery-based mental health workers’ Experiences and beliefs. Adv Ment Health (2019) 17(2):98–109. doi: 10.1080/18387357.2018.1493346

[50] Van Wert MJ Malik M Memel B Moore R Buccino D Hackerman F. Provider perceived barriers and facilitators to integrating routine outcome monitoring into practice in an urban community psychiatry clinic: A mixed-methods quality improvement project. J Eval Clin Pract (2021) 27(4):767–75. doi: 10.1111/jep.13457 32790131

[51] Tickle A Cheung N Walker C. Professionals’ Perceptions of the mental health recovery star. Ment Health Rev J (2013) 18(4):194–203. doi: 10.1108/MHRJ-04-2013-0015

[52] Dowrick C Leydon GM McBride A Howe A Burgess H Clarke P. Patients’ and doctors’ Views on depression severity questionnaires incentivised in Uk quality and outcomes framework: qualitative study. BMJ (2009) 338:663–72. doi: 10.1136/bmj.b663 19299474

[53] Boyce MB Browne JP Greenhalgh J. The experiences of professionals with using information from patient-reported outcome measures to improve the quality of healthcare: A systematic review of qualitative research. BMJ Qual Saf (2014) 23(6):508–18. doi: 10.1136/bmjqs-2013-00252 24505110

[54] Gordon SE Ellis PM. Recovery of evidence-based practice. Int J Ment Health Nurs (2013) 22(1):3–14. doi: 10.1111/j.1447-0349.2012.00835.x 22830603

[55] Börjesson S Boström PK. “I want to know what it is used for”: clients’ Perspectives on completing a routine outcome measure (Rom) while undergoing psychotherapy. Psychother Res (2020) 30(3):337–47. doi: 10.1080/10503307.2019.1630780 31198093

[56] Solstad SM Castonguay LG Moltu C. Patients' Experiences with routine outcome monitoring and clinical feedback systems: A systematic review and synthesis of qualitative empirical literature. Psychother Res (2019) 29(2):157–70. doi: 10.1080/10503307.2017.1326645 28523962

