# Supplementary material for: Feasibility of Recovery Assessment Scale – Domains and Stages (RAS-DS) for everyday mental health practice
Source: Front Psychiatry. 2024 Feb 9;15:1256092. doi: 10.3389/fpsyt.2024.1256092 (PMC10884109; doi:10.3389/fpsyt.2024.1256092)
Supplement: Supplementary file 1 [file DataSheet_1.docx]

Survey items

**Feasibility of the Recovery Assessment Scale – Domains and Stages (RAS-DS) in everyday clinical practice**

Please state your gender / gender identity (if you prefer not to say, please write "prefer not to say")

__________________________________

Please select your age:

- Under 20 years
- 20 to 29 years
- 30 to 39 years
- 40 to 49 years
- 50 to 59 years
- 60 to 69 years
- 70 years or older
- Prefer not to say

How long have you worked in mental health?

- Less than 1 year
- 1 to 2 years
- 3 to 5 years
- 6 to 10 years
- 11 to 20 years
- Over 20 years
- Prefer not to say

What best describes your current role:

- Mental health worker / clinician
- Consumer worker / peer worker
- Generalist health worker
- Other

Do you have qualifications in any of the following:

- counselling
- nursing
- occupational therapy
- psychiatry
- psychology
- social work
- other
- If 'other' above, please describe role or profession:

__________________________________

Which setting/s do you currently work in? (select all that apply)

- Mental health services - Acute inpatient / hospital
- Mental Health Services - Inpatient Rehabilitation
- Mental Health Services - Community based
- Drug and Alcohol / Addiction services
- Other
- Not applicable
- If 'other' or 'not applicable' above, please describe:

__________________________________

What age group are the people you work with? (select all that apply)

- Children (0 to 12)
- Adolescents (12 to 18)
- Youth / Early intervention services (approx. 12 to 25)
- Adult services (18 to 65)
- Older adult services (65 and over)
- Other
- Not applicable
- If 'other' above, please describe:

__________________________________

Do you currently use RAS-DS in your work?

- Yes
- No

Have you used RAS-DS in the past?

- Yes
- No

If you have previously used the RAS-DS but stopped using it, please briefly explain why you decided to stop using it below

__________________________________________

If you have not used the RAS-DS in your work previously, please describe why you decided not to use it in the space below

__________________________________________

What country do you work in?

__________________________________

Which state / province do you work in?

__________________________________

What language/s do you use RAS-DS in?

- English
- Arabic
- Chinese (Traditional)
- Chinese (Simplified)
- Dinka
- Dutch
- Farsi
- Icelandic
- Indonesian
- Italian
- Korean
- Spanish
- Thai
- Vietnamese
- Other

What other languages do you use the RAS-DS in:

__________________________________

Why do you use RAS-DS? (select all that apply)

- Because the use of the RAS-DS is mandated by the service I work in
- Because I find it valuable to support my practice
- Other

For "other" above - please specify:

__________________________________

Please select the MAIN way in which you use the RAS-DS (select one answer only):

- To guide my work with the people I work with
- For research / quality improvement
- To monitor service outcomes
- Other
- [I don't know / not sure what the RAS-DS is used for]

For "other" above - please specify:

__________________________________

Are there any other ways or reasons why you use RAS-DS (select all that apply):

To guide my work with the people I work with

- For research / quality improvement
- To monitor service outcomes
- Other
- N/A - I only use the RAS-DS for the purpose listed in the previous question.

For "other" above - please specify:

__________________________________

Overall, how often do you use RAS-DS in your work?

- Less than once a month
- At least once a month
- At least once every two weeks
- At least once a week

Typically, how frequently do you use RAS-DS with each person you work with?

- Just once
- Less than once a year
- About once a year
- About once every six moths
- About once every three months
- About once a month
- More often than once a month

Optional: Please add any detail to explain your answers above in terms of the frequency of your use

of the RAS-DS.

__________________________________________

What proportion of people who you work with do you use the RAS-DS with?

- Over 90%
- Between 75% and 90%
- Between 50% and 75%
- Between 25% and 50%
- Less than 25%

What are some of the reasons / factors you consider when decided to use / not use the RAS-DS (optional):

__________________________________

Overall, how useful would you rate the RAS-DS?

- Not useful at all
- Only slightly useful
- Moderately useful
- Quite useful
- Very useful

Please explain your rating above:

__________________________________________

After completing the RAS-DS, how do you use the results? Please describe below.

__________________________________________

From your perspective, what are some of the most useful / positive aspects of using the RAS-DS?

__________________________________________

From your perspective, what are some of the least useful / negative aspects of using the RAS-DS?

__________________________________________

Are there any aspects / areas / items missing from the RAS-DS? If so, what are these?

__________________________________________

In the table below, please indicate your level of agreement with each of the statements. There is a section for recording comments after each question, if needed.

|  | Strongly disagree | Disagree | Neutral | Agree | Strongly agree | Don’t know |
| --- | --- | --- | --- | --- | --- | --- |
| The RAS-DS is too long for people I work with complete | 🞐 | 🞐 | 🞐 | 🞐 | 🞐 | 🞐 |
| Optional: please comment on the rating above: | | | | | | |
|  | Strongly disagree | Disagree | Neutral | Agree | Strongly agree | Don’t know |
| The training required to administer the RAS-DS is minimal | 🞐 | 🞐 | 🞐 | 🞐 | 🞐 | 🞐 |
| Optional: please comment on the rating above: | | | | | | |
|  | Strongly disagree | Disagree | Neutral | Agree | Strongly agree | Don’t know |
| It is easy to score the RAS-DS | 🞐 | 🞐 | 🞐 | 🞐 | 🞐 | 🞐 |
| Optional: please comment on the rating above: | | | | | | |
|  | Strongly disagree | Disagree | Neutral | Agree | Strongly agree | Don’t know |
| It is easy interpret results from the RAS-DS | 🞐 | 🞐 | 🞐 | 🞐 | 🞐 | 🞐 |
| Optional: please comment on the rating above: | | | | | | |
|  | Strongly disagree | Disagree | Neutral | Agree | Strongly agree | Don’t know |
| The RAS-DS has too many items / questions | 🞐 | 🞐 | 🞐 | 🞐 | 🞐 | 🞐 |
| Optional: please comment on the rating above: | | | | | | |
|  | Strongly disagree | Disagree | Neutral | Agree | Strongly agree | Don’t know |
| It is easy to access the RAS-DS | 🞐 | 🞐 | 🞐 | 🞐 | 🞐 | 🞐 |
| Optional: please comment on the rating above: | | | | | | |
|  | Strongly disagree | Disagree | Neutral | Agree | Strongly agree | Don’t know |
| The purpose of the RAS-DS is clear and relevant for the people I work with | 🞐 | 🞐 | 🞐 | 🞐 | 🞐 | 🞐 |
| Optional: please comment on the rating above: | | | | | | |
|  | Strongly disagree | Disagree | Neutral | Agree | Strongly agree | Don’t know |
| The wording of the items in the RAS-DS is easy for people I work with to understand | 🞐 | 🞐 | 🞐 | 🞐 | 🞐 | 🞐 |
| Optional: please comment on the rating above: | | | | | | |
|  | Strongly disagree | Disagree | Neutral | Agree | Strongly agree | Don’t know |
| The RAS-DS is culturally appropriate for the consumers I work with | 🞐 | 🞐 | 🞐 | 🞐 | 🞐 | 🞐 |
| Optional: please comment on the rating above: | | | | | | |
|  | Strongly disagree | Disagree | Neutral | Agree | Strongly agree | Don’t know |
| People I work with are able to complete RAS-DS with me or on their own if they choose | 🞐 | 🞐 | 🞐 | 🞐 | 🞐 | 🞐 |
| Optional: please comment on the rating above: | | | | | | |
|  | Strongly disagree | Disagree | Neutral | Agree | Strongly agree | Don’t know |
| The areas covered by the RAS-DS are meaningful and important to the people I work with | 🞐 | 🞐 | 🞐 | 🞐 | 🞐 | 🞐 |
| Optional: please comment on the rating above: | | | | | | |
|  | Strongly disagree | Disagree | Neutral | Agree | Strongly agree | Don’t know |
| Having consumers complete the RAS-DS gives me a clearer understanding of them and their perspectives | 🞐 | 🞐 | 🞐 | 🞐 | 🞐 | 🞐 |
| Optional: please comment on the rating above: | | | | | | |
|  | Strongly disagree | Disagree | Neutral | Agree | Strongly agree | Don’t know |
| The RAS-DS helps people I work with to participate more in decision-making, treatment planning and tracking their progress | 🞐 | 🞐 | 🞐 | 🞐 | 🞐 | 🞐 |
| Optional: please comment on the rating above: | | | | | | |
|  | Strongly disagree | Disagree | Neutral | Agree | Strongly agree | Don’t know |
| The RAS-DS helps promote discussion | 🞐 | 🞐 | 🞐 | 🞐 | 🞐 | 🞐 |
| Optional: please comment on the rating above: | | | | | | |

About what proportion of consumers who you ask to complete the RAS-DS agree to complete it?

- Over 90%
- Between 75% and 90%
- Between 50% and 75%
- Between 25% and 50%
- Less than 25%

When consumers complete the RAS-DS, about how many items are missed / skipped?

- None, usually consumers complete all items
- Usually only one or two skipped
- Three to five
- Six to ten
- 11 to 20
- Over 20

In your experience, which items are skipped most frequently?

Do you think it would it be useful if we added a discussion or comments section at the end of the RAS-DS to ask people if they have is anything else important to them that was not covered in the RAS-DS?

- No
- Yes, to a small degree
- Yes, to a moderate degree
- Yes, to a large degree
- [Don't know]

If no, please provide an explanation (optional)

__________________________________________

If yes, please provide an explanation (optional)

__________________________________________

Do you believe the RAS-DS helps you to be more recovery-oriented in your practice?

- No
- Yes, to a small degree
- Yes, to a moderate degree
- Yes, to a large degree
- [Don’t know]

If no, please provide an explanation (optional)

__________________________________________

If yes, please provide an explanation (optional)

__________________________________________

We would appreciate any other comment you have about using the RAS-DS in your work

__________________________________________

**Thank you for completing this survey**
